# Supplementary material for: Hot spot prediction in protein-protein interactions by an ensemble system
Source: BMC Syst Biol. 2018 Dec 31;12(Suppl 9):132. doi: 10.1186/s12918-018-0665-8 (PMC6311905; doi:10.1186/s12918-018-0665-8)
Supplement: Supplementary file 2 — All relevant MATLAB code. A simple Matlab implement of our predictor. (ZIP 19,742 kb) [file 12918_2018_665_MOESM2_ESM.zip › Additional file 2/Read ASA/ASA website/dbMPIKT/4KRMB.html]

 NetSurfP - Protein Surface Accessibility and Secondary Structure Predictions

  

|  |  |  |
| --- | --- | --- |
|  |  | NetSurfP - Protein Surface Accessibility and Secondary Structure Predictions  Technical University of Denmark |

  


---

```
# For publication of results, please cite:
# A generic method for assignment of reliability scores applied to solvent accessibility predictions.
# Bent Petersen, Thomas Nordahl Petersen, Pernille Andersen, Morten Nielsen and Claus Lundegaard
# BMC Structural Biology 2009, 9:51 doi:10.1186/1472-6807-9-51
#
# Column 1: Class assignment - B for buried or E for Exposed - Threshold: 25% exposure, but not based on RSA
# Column 2: Amino acid
# Column 3: Sequence name
# Column 4: Amino acid number
# Column 5: Relative Surface Accessibility - RSA
# Column 6: Absolute Surface Accessibility
# Column 7: Z-fit score for RSA prediction
# Column 8: Probability for Alpha-Helix
# Column 9: Probability for Beta-strand
# Column 10: Probability for Coil
E Q  4KRMB                  1    0.744 132.896   1.161   0.003   0.003   0.994
B V  4KRMB                  2    0.219  33.614  -0.346   0.004   0.616   0.381
E K  4KRMB                  3    0.407  83.720   1.079   0.002   0.816   0.182
B L  4KRMB                  4    0.166  30.321   0.521   0.002   0.816   0.182
E E  4KRMB                  5    0.366  63.940   0.437   0.021   0.756   0.223
B E  4KRMB                  6    0.241  42.015   0.623   0.004   0.514   0.481
E S  4KRMB                  7    0.377  44.208  -0.459   0.020   0.205   0.775
E G  4KRMB                  8    0.451  35.525  -1.799   0.018   0.088   0.893
E G  4KRMB                  9    0.503  39.570  -2.262   0.018   0.047   0.935
E G  4KRMB                 10    0.497  39.130  -1.706   0.019   0.141   0.840
E S  4KRMB                 11    0.370  43.317  -0.774   0.022   0.359   0.619
E V  4KRMB                 12    0.293  45.065   0.466   0.022   0.359   0.619
E Q  4KRMB                 13    0.399  71.208   0.991   0.020   0.205   0.775
E T  4KRMB                 14    0.465  64.495  -0.626   0.018   0.047   0.935
E G  4KRMB                 15    0.590  46.457  -2.087   0.005   0.045   0.951
E G  4KRMB                 16    0.408  32.086  -0.879   0.004   0.085   0.910
E S  4KRMB                 17    0.390  45.685  -0.663   0.004   0.420   0.576
B L  4KRMB                 18    0.151  27.557   0.672   0.002   0.816   0.182
E R  4KRMB                 19    0.391  89.539   0.770   0.001   0.900   0.099
B L  4KRMB                 20    0.070  12.817   0.543   0.001   0.900   0.099
B T  4KRMB                 21    0.138  19.085   0.804   0.001   0.900   0.099
B C  4KRMB                 22    0.040   5.560   0.173   0.001   0.900   0.099
B A  4KRMB                 23    0.279  30.779   0.598   0.001   0.900   0.099
B A  4KRMB                 24    0.086   9.444  -0.304   0.003   0.718   0.279
B S  4KRMB                 25    0.180  21.073  -0.671   0.004   0.420   0.576
B G  4KRMB                 26    0.221  17.424  -1.802   0.004   0.197   0.799
B R  4KRMB                 27    0.248  56.678  -0.725   0.005   0.262   0.733
B T  4KRMB                 28    0.142  19.668  -0.813   0.004   0.514   0.481
B S  4KRMB                 29    0.111  13.068  -0.939   0.021   0.451   0.528
B R  4KRMB                 30    0.208  47.540  -0.972   0.021   0.279   0.699
B S  4KRMB                 31    0.294  34.457  -1.167   0.018   0.088   0.893
B Y  4KRMB                 32    0.102  21.883   0.044   0.019   0.141   0.840
B G  4KRMB                 33    0.071   5.556  -0.013   0.021   0.451   0.528
B M  4KRMB                 34    0.024   4.802   0.634   0.021   0.756   0.223
B G  4KRMB                 35    0.047   3.699  -0.069   0.018   0.846   0.136
B W  4KRMB                 36    0.032   7.816   0.530   0.018   0.846   0.136
B F  4KRMB                 37    0.036   7.165   0.273   0.018   0.846   0.136
B R  4KRMB                 38    0.108  24.663   0.608   0.021   0.756   0.223
B Q  4KRMB                 39    0.091  16.324   0.244   0.022   0.359   0.619
B A  4KRMB                 40    0.205  22.646   0.816   0.005   0.045   0.951
E P  4KRMB                 41    0.343  48.700   0.656   0.005   0.015   0.979
E G  4KRMB                 42    0.546  42.986  -1.565   0.018   0.019   0.964
E K  4KRMB                 43    0.406  83.411   0.564   0.018   0.019   0.964
E E  4KRMB                 44    0.458  79.978  -0.307   0.018   0.088   0.893
B R  4KRMB                 45    0.158  36.182   0.928   0.005   0.336   0.660
B E  4KRMB                 46    0.103  17.942  -0.127   0.003   0.718   0.279
B F  4KRMB                 47    0.050   9.935   0.205   0.001   0.900   0.099
B V  4KRMB                 48    0.026   4.042   0.829   0.001   0.959   0.040
B S  4KRMB                 49    0.017   2.016   0.050   0.001   0.959   0.040
B G  4KRMB                 50    0.080   6.327  -0.867   0.001   0.959   0.040
B I  4KRMB                 51    0.023   4.310   0.918   0.001   0.959   0.040
B S  4KRMB                 52    0.138  16.174   0.848   0.002   0.816   0.182
B W  4KRMB                 53    0.279  67.075  -0.937   0.005   0.336   0.660
E R  4KRMB                 54    0.546 125.103  -1.537   0.005   0.015   0.979
E G  4KRMB                 55    0.536  42.167  -2.582   0.018   0.019   0.964
E D  4KRMB                 56    0.560  80.710  -2.051   0.018   0.019   0.964
E S  4KRMB                 57    0.461  54.053  -0.529   0.018   0.088   0.893
B T  4KRMB                 58    0.281  39.016   0.125   0.021   0.279   0.699
E G  4KRMB                 59    0.400  31.504   0.151   0.021   0.279   0.699
B Y  4KRMB                 60    0.199  42.484   0.351   0.066   0.296   0.638
B A  4KRMB                 61    0.180  19.803  -0.448   0.052   0.084   0.864
E D  4KRMB                 62    0.629  90.596  -0.509   0.053   0.043   0.903
E S  4KRMB                 63    0.385  45.110   0.103   0.052   0.084   0.864
B V  4KRMB                 64    0.077  11.881  -0.406   0.020   0.205   0.775
E K  4KRMB                 65    0.382  78.639   0.846   0.020   0.205   0.775
B G  4KRMB                 66    0.205  16.165  -1.434   0.005   0.336   0.660
B R  4KRMB                 67    0.224  51.182   0.999   0.003   0.718   0.279
B F  4KRMB                 68    0.052  10.517  -0.085   0.001   0.900   0.099
B T  4KRMB                 69    0.122  16.880   0.826   0.001   0.959   0.040
B I  4KRMB                 70    0.040   7.326   0.805   0.001   0.900   0.099
B S  4KRMB                 71    0.126  14.791   0.461   0.002   0.816   0.182
B R  4KRMB                 72    0.241  55.281   0.610   0.022   0.552   0.426
B D  4KRMB                 73    0.243  35.045  -0.261   0.022   0.359   0.619
E N  4KRMB                 74    0.446  65.265   0.147   0.018   0.088   0.893
B A  4KRMB                 75    0.238  26.206  -1.854   0.018   0.047   0.935
E K  4KRMB                 76    0.517 106.244   0.900   0.056   0.142   0.802
E N  4KRMB                 77    0.267  39.060  -0.216   0.066   0.296   0.638
B T  4KRMB                 78    0.173  23.953   0.976   0.023   0.655   0.322
B V  4KRMB                 79    0.078  11.927  -0.495   0.018   0.846   0.136
B D  4KRMB                 80    0.165  23.834   0.353   0.018   0.846   0.136
B L  4KRMB                 81    0.051   9.265   0.730   0.018   0.846   0.136
B Q  4KRMB                 82    0.150  26.808  -0.175   0.021   0.756   0.223
B M  4KRMB                 83    0.084  16.788  -0.599   0.022   0.552   0.426
E N  4KRMB                 84    0.351  51.372  -0.310   0.021   0.279   0.699
E S  4KRMB                 85    0.379  44.419  -0.668   0.018   0.088   0.893
B L  4KRMB                 86    0.092  16.754   0.024   0.018   0.047   0.935
E K  4KRMB                 87    0.478  98.386   1.169   0.018   0.047   0.935
E P  4KRMB                 88    0.357  50.602  -0.606   0.053   0.043   0.903
E E  4KRMB                 89    0.446  77.846  -0.560   0.053   0.043   0.903
B D  4KRMB                 90    0.167  24.007  -0.978   0.053   0.043   0.903
B T  4KRMB                 91    0.109  15.063   0.305   0.066   0.296   0.638
B A  4KRMB                 92    0.039   4.254  -0.434   0.021   0.756   0.223
B I  4KRMB                 93    0.069  12.802   1.012   0.018   0.846   0.136
B Y  4KRMB                 94    0.036   7.693   0.871   0.018   0.846   0.136
B Y  4KRMB                 95    0.048  10.258   0.980   0.018   0.846   0.136
B C  4KRMB                 96    0.015   2.064   1.596   0.021   0.756   0.223
B A  4KRMB                 97    0.027   3.008   0.260   0.022   0.552   0.426
B A  4KRMB                 98    0.121  13.279   0.088   0.022   0.359   0.619
B A  4KRMB                 99    0.241  26.558  -0.289   0.020   0.205   0.775
E A  4KRMB                100    0.313  34.526  -0.821   0.018   0.088   0.893
E G  4KRMB                101    0.353  27.750  -1.760   0.018   0.047   0.935
E S  4KRMB                102    0.425  49.845  -2.216   0.018   0.088   0.893
E A  4KRMB                103    0.365  40.245  -2.576   0.018   0.088   0.893
B W  4KRMB                104    0.285  68.422  -2.276   0.018   0.088   0.893
B Y  4KRMB                105    0.292  62.336  -2.332   0.018   0.047   0.935
B G  4KRMB                106    0.276  21.690  -2.233   0.018   0.047   0.935
B T  4KRMB                107    0.282  39.100  -1.691   0.018   0.047   0.935
B L  4KRMB                108    0.285  52.238  -1.756   0.018   0.047   0.935
B Y  4KRMB                109    0.244  52.207  -1.179   0.018   0.047   0.935
B E  4KRMB                110    0.107  18.710  -1.198   0.018   0.088   0.893
B Y  4KRMB                111    0.051  10.856  -0.046   0.019   0.141   0.840
B D  4KRMB                112    0.079  11.355  -0.570   0.019   0.141   0.840
B Y  4KRMB                113    0.062  13.228  -0.275   0.020   0.205   0.775
B W  4KRMB                114    0.043  10.245  -0.405   0.019   0.141   0.840
B G  4KRMB                115    0.040   3.117  -1.438   0.019   0.141   0.840
B Q  4KRMB                116    0.146  26.129   0.147   0.018   0.088   0.893
B G  4KRMB                117    0.096   7.563  -1.351   0.005   0.262   0.733
B T  4KRMB                118    0.060   8.391   0.211   0.004   0.514   0.481
B Q  4KRMB                119    0.214  38.167   0.331   0.002   0.816   0.182
B V  4KRMB                120    0.036   5.518   0.497   0.011   0.918   0.071
B T  4KRMB                121    0.081  11.221   0.537   0.011   0.918   0.071
B V  4KRMB                122    0.066  10.129   0.364   0.018   0.846   0.136
B S  4KRMB                123    0.172  20.217  -0.191   0.004   0.514   0.481
B S  4KRMB                124    0.228  26.710  -1.060   0.020   0.205   0.775
E A  4KRMB                125    0.396  43.683  -1.743   0.018   0.047   0.935
E L  4KRMB                126    0.441  80.747  -0.134   0.018   0.047   0.935
E E  4KRMB                127    0.402  70.142  -0.782   0.018   0.047   0.935
E H  4KRMB                128    0.570 103.738  -0.523   0.018   0.019   0.964
E H  4KRMB                129    0.390  71.032  -1.263   0.005   0.015   0.979
B H  4KRMB                130    0.271  49.331  -1.248   0.005   0.045   0.951
E H  4KRMB                131    0.573 104.229  -0.330   0.019   0.141   0.840
E H  4KRMB                132    0.542  98.572  -0.580   0.019   0.141   0.840
E H  4KRMB                133    0.721 131.132  -1.528   0.003   0.003   0.994

Explain the output.  Go back.
```

---
